# CT Perfusion Characteristics Identify Metastatic Sites in Liver

**DOI:** 10.1155/2015/120749

**Published:** 2015-10-05

**Authors:** Yuan Wang, Brian P. Hobbs, Chaan S. Ng

**Affiliations:** ^1^Department of Biostatistics, The University of Texas MD Anderson Cancer Center, Houston, TX 77030, USA; ^2^Department of Diagnostic Radiology, The University of Texas MD Anderson Cancer Center, Houston, TX 77030, USA

## Abstract

Tissue perfusion plays a critical role in oncology because growth and migration of cancerous cells require proliferation of new blood vessels through the process of tumor angiogenesis. Computed tomography (CT) perfusion is an emerging functional imaging modality that measures tissue perfusion through dynamic CT scanning following intravenous administration of contrast medium. This noninvasive technique provides a quantitative basis for assessing tumor angiogenesis. CT perfusion has been utilized on a variety of organs including lung, prostate, liver, and brain, with promising results in cancer diagnosis, disease prognostication, prediction, and treatment monitoring. In this paper, we focus on assessing the extent to which CT perfusion characteristics can be used to discriminate liver metastases from neuroendocrine tumors from normal liver tissues. The neuroendocrine liver metastases were analyzed by distributed parameter modeling to yield tissue blood flow (BF), blood volume (BV), mean transit time (MTT), permeability (PS), and hepatic arterial fraction (HAF), for tumor and normal liver. The result reveals the potential of CT perfusion as a tool for constructing biomarkers from features of the hepatic vasculature for guiding cancer detection, prognostication, and treatment selection.

## 1. Introduction

Tumor angiogenesis is the process of proliferation of new blood vessel during the growth and spread of tumors. Quantification of this process provides assessment of tumor growth at early stages and can provide prognostic, predictive, and surrogate power. In general, the tumor vessels increase in density over time, and they also function abnormally. For example, tumor vessels tend to be less leaky and more easily compressed [1]. This limits the traditional morphological imaging techniques, which emphasize the quantification simply of structural information. Computed tomography (CT) perfusion is an emerging functional imaging modality that measures characteristics pertaining to the vascular perfusion of tissues. Perfusion imaging, which provides a quantitative basis for assessing vasculature heterogeneity induced by tumor angiogenesis, has much potential in cancer detection, disease prognostication, and treatment monitoring. Many other imaging technologies such as magnetic resonance (MR) imaging, ultrasound (US), and positron emission tomography (PET) are being developed to measure perfusion characteristics [2]. Compared to these techniques, CT perfusion is most widely used because it can easily be integrated into routine CT imaging without additional technical training. Moreover, the wide availability of standardized CT imaging makes CT perfusion more accessible compared with other tools.

CT perfusion has been utilized in a number of organs including prostate, colorectal, liver, head and neck, and lung [3]. By providing functional information about the microenvironment surrounding tumor tissue, CT perfusion can assist cancer diagnosis, treatment prognostication, prediction, and monitoring. It has shown promising results for diagnosing primary or metastatic tumors [4]. It also enables assessment of tumor vascularity and perfusion changes that result from chemotherapy and radiation therapy. Moreover, it has been suggested that tumors with high vascularity tend to be more aggressive and respond poorly to chemotherapy and radiation therapy [5].

In this paper, we review the CT perfusion technology and discuss its application to a case study to assess the extent to which CT perfusion characteristics can be used to discriminate liver metastases from neuroendocrine tumors from normal liver tissues. Liver is the second most common metastatic site after lymph nodes. Early detection is the key for successful treatment of liver tumors. Currently, diagnosis and treatment monitoring of liver cancer is mostly performed using morphological imaging such as MR, CT, and US. With the introduction of molecularly targeted therapies, these approaches may not fully access tumor information. Assessment of perfusion provides functional information of the tissue microenvironment. A variety of perfusion parameters have been produced to characterize tissue perfusion. The most frequently encountered parameters are blood flow (BF), the rate of blood passing through the vasculature in a tissue region, measured in mL/min/100 g; blood volume (BV), the volume of blood that is actually flowing within the vasculature in a tissue region, measured in mL/100 g; mean transit time (MTT), the average time for blood traversing from the arterial input to the venous outlet, measured in seconds. Both BF and BV correlate with the density of microvessels. Increased number of vessels would increase BF and BV correspondingly. MTT reflects perfusion pressure in a way that higher perfusion pressure pushes blood traveling at a higher velocity and results in a shorter mean transit time. Permeability surface area product (PS) is another widely studied perfusion parameter; it is the product of permeability and the total surface area of capillary endothelium in a unit mass of tissue, measured in units of mL/min/100 g. PS is a surrogate measure of vascular leakiness and it reflects the flux of solutes from blood plasma to the interstitial space. In the study of liver, hepatic arterial fraction (HAF) is another important parameter. The liver has a dual system of blood supply from the hepatic artery and the portal vein. HAF, which is the proportion of liver blood supplied by the hepatic artery, provides a measure of liver perfusion derived from arterial rather than portal blood.

Existing studies have shown that perfusion characteristics correlate well with the presence of tumor vessels [6]. The relationship between perfusion parameters and tumor angiogenesis is complex [3, 6, 7]. The growth and spread of tumor rely heavily on proliferation of new blood vessels. The increased density of microvessels will result in increased tumor perfusion and consequently changes in the distributions of perfusion parameters [8]. Guyennon et al. [9] have demonstrated that, compared with healthy tissues, metastatic neuroendocrine tumors had significantly higher blood flow, blood volume, and permeability surface area product and significantly shorter MTT. Reiner et al. [10] have shown significantly increased hepatic arterial perfusion and decreased portal venous perfusion in colorectal cancer. Moreover, the difference in permeability of malignant and normal tissues varies with the target organ. Permeability levels for brain tumor are considerably higher, while in other organs the difference is generally lower in comparison to brain [8]. In general, tumor vessels tend to have incomplete basement membranes which lead to increased permeability and leakage space. Our study yielded statistically significant evidence to suggest that four perfusion characteristics, BF, MTT, PS, and HAF, effectively discriminate between ROIs that contain neuroendocrine metastases from sites containing healthy liver tissues.

## 2. Materials and Methods

**Figure 1:**
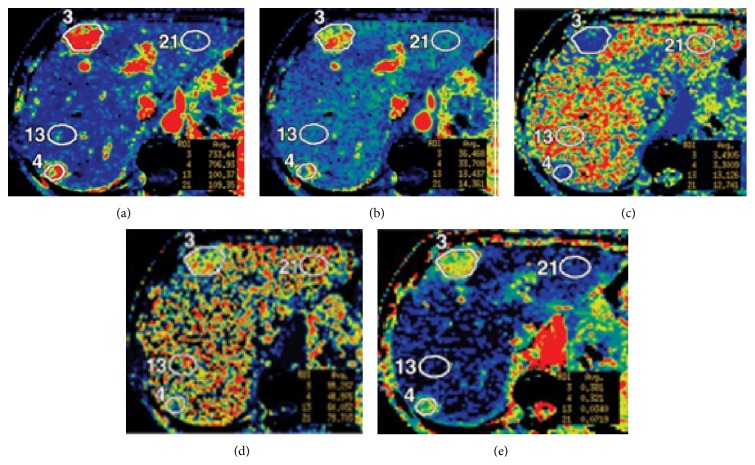
Maps for BF, BV, MTT, PS, and HAF at acquisition durations of 590 seconds ((a)–(e), resp.). BF is expressed in mL/min per 100 g; BV, in mL/100 g; MTT, in seconds; and PS, in mL/min per 100 g.

The study focused on patients with neuroendocrine liver metastases who underwent CT perfusion of a target lesion in the liver, in which malignancy was determined clinically or radiologically. The study collected data between April 2007 and September 2009 on 16 patients. CT perfusion images (Figure 1) were obtained from a dual phase protocol spanning a duration of 590 seconds (s). The images were obtained with a 64-row multidetector CT scanner (VCT, GE Healthcare, Waukesha, WI). The scans were obtained in two phases: Phase 1, cine acquisition during a breath-hold, followed by Phase 2, consisting of intermittent short breath-hold helical scans. The dataset analyzed here consisted of fifty-nine 8-slice cine images temporally sampled at 0.5 s from the Phase 1 acquisition, together with eight anatomically matched images from the Phase 2 acquisition. Five perfusion characteristics were acquired: blood flow (BF), blood volume (BV), mean transit time (MTT), permeability surface area product (PS), and hepatic arterial fraction (HAF). Figure 1 illustrates the five CTp characteristics obtained for a single patient at the end of the acquisition duration. Our analysis used the average BF, BV, MTT, PS, and HAF values obtained at acquisition time 590 s, a duration that was shown to yield stable acquisition in the liver. The values of the CTp characteristics were averaged over all 8 slices of the acquired CT perfusion images. More information of the study is available in [11].

### 2.1. Acquisition of CT Perfusion

Typically, CT perfusion acquisition requires intravenous injection of iodinated contrast medium and repeated CT data from the target tissue. The contrast medium passes through human body within the intravascular space and the extravascular extracellular space. The tissue enhancement is proportional to the local concentration of the contrast medium at any given time. By tracking the local concentration of the contrast medium in the tissue over time, the time-intensity curve can be observed for any region of interest (ROI). The distribution of contrast medium largely reveals blood flow and perfusion. Different approaches have been developed to estimate perfusion parameters. One approach is the distributed parameter model, which uses the deconvolution of the tissue and vascular time-intensity curves [12]. For the distributed parameter model, the tissue and vascular time-intensity curves over the whole acquisition are used for calculating perfusion parameters.

The quality of the resulting perfusion data depends on the manner in which the data is acquired. When specifying an acquisition protocol, investigators must determine several factors that could affect the quality of the resultant perfusion measurements. In particular, these include duration of scan acquisition, temporal sampling frequency, and the preenhancement set point. Additionally, some imaging preprocessing, such as motion correction, may be necessary.

#### 2.1.1. Duration of Scan

In current clinical applications of CT perfusion in liver, the durations of acquisitions vary between half a minute and 10 minutes. Reduced acquisition duration offers less radiation exposure but may compromise the quality of the CT perfusion parameter values. Ng et al. [13] have shown in a study of lung cancer that CT perfusion parameter values derived from deconvolution modeling can be markedly affected by the acquisition duration. Ng et al. [11] described that determination of appropriate and sufficiently long acquisition durations depends on the degree of confidence required for those parameters. Moreover, the results can vary depending on the specific parameter of interest. 160 s was required to obtain at least low confidence of stability for any of the CT perfusion parameters in liver. PS requires longer acquisition time when compared with BF, BV, MTT, and HAF.

#### 2.1.2. Sampling Interval

Another factor that affects the overall radiation exposure is the frequency of CT scan, or the sampling interval (SI). The CT images are acquired at relatively high temporal sampling frequencies, typically with a temporal sampling interval of 1 second or less. The overall radiation exposure could be reduced if the temporal SI could be increased. Ng et al. [14] investigated the effect of SIs on CT perfusion parameter values in liver tumors and normal tissue and, in particular, one that implements the dual vascular (arterial and portal venous) inputs that are relevant to this particular organ. They have shown that increasing SIs beyond 1 second yielded significantly different CT perfusion parameter values when compared with the reference values at SI of 0.5 seconds.

#### 2.1.3. Preenhancement Set Point

Perfusion parameters calculated using the distributed parameter model also depend on the input tissue and vascular time-intensity curves. The preenhancement set point (*T*_1_), that is, the time when the arterial concentration first begins to rise, is one crucial factor in defining these time-intensity curves. This is a user-defined variable and is inevitably subject to observer variation. There have been a few studies that have investigated the potential effects of the positioning of *T*_1_ on CT perfusion parameter values using distributed parameter modeling. Sanelli et al. [15] have shown that, in the study of brain, variations in the delineation of *T*_1_ could lead to significant change in the resultant CT perfusion parameter values. Ng et al. [16] compared varying preenhancement displacements in a study of liver metastases and showed that the absolute values of CT perfusion parameters were affected by the positioning of *T*_1_. Moreover, positive displacements in *T*_1_ greater than or equal to 1.0 second were more deleterious than corresponding negative displacements, when comparing the impact on CTp values in relation to the reference.

### 2.2. Statistical Analysis

Receiver operating characteristics (ROCs) were computed to evaluate the extent to which perfusion characteristics acquired using CT can be used to discriminate between ROIs that contain liver metastases from those with healthy liver tissues. Univariate logistic regression analysis was implemented to estimate the extent to which the odds that given ROI contains a metastatic lesion change as function of each of the five characteristics separately. For each characteristic, two-sided Wald tests were applied to test for a trend. We report the resulting *p* values as well as the corresponding areas under the ROC curves (AUCs). Bonferroni correction was applied to adjust for multiple comparisons among the five comparisons. A *p* value of 0.01 was used to confer statistical significance. Additionally, multiple logistic regression was used to evaluate the extent to which discrimination could be improved in multivariate analysis. All plots and analyses were performed using the statistical software R 3.0.1 (R Foundation, Vienna, Austria).

## 3. Results

**Figure 2:**
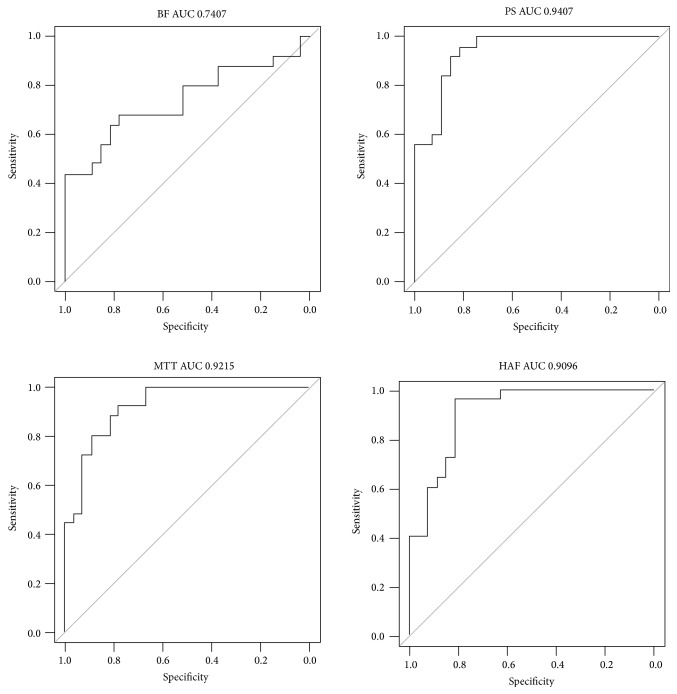
ROCs for classifying liver metastases from normal tissue using perfusion parameters BF, PS, MTT, and HAF with corresponding AUCs: 0.74, 0.94, 0.92, and 0.91, respectively.

Table 1 provides summary statistics for each characteristic for metastatic tumor and normal liver ROIs.  Table 2 provides the *p* values that result from univariate logistic regression analysis as well as the AUCs.  Figure 2 shows the ROCs for classifying liver metastases from normal tissue using perfusion parameters BF, PS, MTT, and HAF, respectively. With the exception of BV, the CT perfusion characteristics were significantly associated with ROI status (tumor versus normal liver). Moreover, MTT, PS, and HAF were highly associated with the presence of a metastatic ROI, with tissues surrounding liver tumors exhibiting significantly elevated HAF and decreased MTT and PS. PS demonstrated the highest utility for discriminating ROIs with tumor from normal liver ROIs with an AUC of 0.94 on univariate analysis. The AUCs aforementioned were obtained from univariate analysis of each characteristic. Our ability to discriminate tumor from normal ROIs was improved using multivariate logistic regression analysis based on all five perfusion parameters, AUC = 0.97.

The aforementioned results and statistical models pertain to analysis of the extent of association between perfusion parameters and pathologic status. Naturally, the results for predicting metastatic sites are attenuated using these approaches. However, owing to the fact that perfusion characteristics tend to be highly correlated among ROIs within a given patient due to shared features of the hepatic vasculature, predictive detection of metastatic sites can be improved using models that account for interparameter and inter-ROI dependence. For example, increased microvessel density often leads to higher blood flow, higher blood volume, and lower MTT. Moreover, the extent of interdependence varies substantially in magnitude and direction between vasculatures surrounding malignant and healthy tissues, providing additional signal for detecting sites where angiogenesis is taking place within the tumor microenvironment. Wang et al. [17] proposed a spatial multivariate Bayesian approach to quadratic discriminant analysis that can be used to predict the status of multiple ROIs simultaneously. The multivariate model was shown to dramatically improve performance for predicting the status of liver ROIs using the perfusion characteristics acquired in our study. In fact, the simultaneous Bayesian method properly predicted the status of every ROI that contains a metastasis.

## 4. Discussion

Over the past two decades, the development of fast CT scanners and the improvement of analysis techniques have made CT perfusion a promising tool for quantitative analysis of tissue perfusion through features that characterize biological processes associated with tumor angiogenesis. Our study suggests that perfusion parameters obtained in liver effectively discriminate between ROIs that contain neuroendocrine metastases from sites containing healthy liver tissues. Moreover, the resulting characteristics are potentially useful for prognostication and staging, since it has been demonstrated that tumors exhibiting high vascularity tend to be more aggressive and respond poorly to chemotherapy and radiation therapy. CT perfusion also offers the potential for quantitative assessment of treatment response since it enables evaluation of tumor vascularity and perfusion changes that occur following chemotherapy and radiation therapy. This promising technology may realize its full potential as a tool for constructing biomarkers from features of the hepatic vasculature for guiding cancer detection, prognostication, and treatment selection through the implementation of multivariate models that leverage the sources of interdependence between parameters and ROIs. Additionally, multivariate modeling enhances the understanding of vascular heterogeneity.

## Figures and Tables

**Table 1:** Summary of raw data by CT perfusion parameter. BF, in mL/min/100 g; BV, in mL/100 g; MTT, in seconds; PS, in mL/min/100 g; HAF, ratio without units.

Parameter	Tumor		Normal liver	
	Median	Interquartile range	Median	Interquartile range
BF	203.6	138.8–361.9	133.50	94.01–174.50
BV	16.61	9.93–23.02	14.58	11.81–18.61
MTT	6.151	5.057–7.116	8.908	7.826–9.470
PS	55.39	42.67–62.71	82.90	74.31–89.35
HAF	0.4336	0.3625–0.5360	0.21070	0.07761–0.28670

**Table 2:** Results of statistical analyses for association between ROI status (metastatic site versus healthy liver tissue) and perfusion parameters. *p* values obtained from logistic regression are provided for each characteristic as well as the corresponding AUC.

	*p* value	AUC
BF	0.00444	0.7407
BV	0.586	0.5793
PS	0.000475	0.9407
MTT	0.000318	0.9215
HAF	0.001201	0.9096
